# Dilemma of Thrombolysis in a patient with high-risk Pulmonary Embolism with severe Thrombocytopenia: A case report

**DOI:** 10.5339/qmj.2023.39

**Published:** 2024-01-13

**Authors:** Phool Iqbal, Mhd Baraa Habib, Ahmed Hatim, Mohammed Alkhatib, Muhammad Abu Bakar, Sunil Hassan Koya, Awni Alshurafa, Habib Ur Rehman

**Affiliations:** 1 Department of Internal Medicine, New York Medical College/Metropolitan Hospital Center, New York, USA; 2 Department of Internal Medicine, Hamad Medical Corporation, Qatar Email: MAbubakar@hamad.qa; 3 Department of Medical ICU, Hamad Medical Corporation, Qatar; 4 Department of Hematology, Hamad Medical Corporation, Qatar

**Keywords:** high-risk PE, severe thrombocytopenia, lymphoma, thrombolysis

## Abstract

Background: Managing a high-risk pulmonary embolism (PE) in a critically ill patient with severe thrombocytopenia can present a challenging dilemma. There is a high risk of fatal bleeding due to anticoagulation in high-risk PE with thrombocytopenia; therefore, risks and benefits are balanced while dealing with such a critical scenario.

Case Report: We present a case of a female patient with thrombocytopenia who was admitted for management of lymphoma. Her hospital course was complicated by high-risk PE, leading to acute respiratory failure and hypotension, necessitating urgent transfer to the medical intensive care unit. She was intubated and placed on mechanical ventilation. Multiple cardiac arrests occurred due to compromised cardiac output from a severely dilated right ventricle on bedside transthoracic echocardiography. As a last resort to save her life in this critical state and severe thrombocytopenia, she was given a half bolus dose of the recommended drug, i.e., 50mg IV of Alteplase. Subsequently, she stabilized and was extubated without any further complications.

Discussion: High-risk PE needs prompt management with anticoagulation to avoid fatal outcomes. However, on the other hand, anticoagulation carries a high risk of bleeding, especially in patients with thrombocytopenia. These challenges prompt a modern perspective in situations where clear guidelines are absent.

Conclusion: We aim to discuss our contemporary clinical practice in managing such a complex case and highlight the need for further studies.

## Introduction

High-risk pulmonary embolism (PE) is an obstruction of the pulmonary arterial tree that exceeds 50% of the cross-sectional area, resulting in severe pulmonary compromise and acute cardiovascular failure due to right ventricular overload.^1^ A definitive diagnosis requires imaging studies such as ventilation-perfusion scanning (VQ scan), contrast pulmonary angiography (CTPA), computed tomographic [CT] angiography, or echocardiography.^2^ The prognosis of PE depends on the severity of clot burden or obstruction.^2^ Low-risk PE typically has a good prognosis with less than 1% mortality, while intermediate high-risk and high-risk PE carry mortality of 5-25% and more than 20%, respectively.^2^ Management includes urgent systemic thrombolysis and anticoagulation if there are no contraindications or surgical/catheter-based embolectomy in selected critical cases with good outcomes. However, the choice of intervention depends on the patient’s clinical status and the available hospital resources.^3,4^ We present a challenging case of high-risk PE leading to multiple cardiac arrests in a female with severe thrombocytopenia in a public healthcare-based facility.

“The authors took consent from the patient and approval from the institution for the publication of this case report (MRC-04-22-143).”

## Case Presentation

A 42-year-old Filipino woman with a recent diagnosis of lymphoma made two months before her presentation to the emergency department (ED) arrived with two episodes of fainting within a day. She reported symptoms of palpitations, lightheadedness, and exertional shortness of breath. Despite her lymphoma diagnosis, she had not attended her follow-up visits. Upon presentation, her initial vital signs indicated a low-grade fever of 37.8°C, a heart rate of 110 beats per minute, a respiratory rate of 24 breaths per minute, and normal room air oxygen saturation of 97%. Physical examination revealed a right cervical mass, multiple enlarged cervical, supraclavicular, and inguinal lymph nodes, and splenomegaly.

An urgent consultation with a hematologist/oncologist was sought for the management of lymphoma. She was subsequently transferred to a local cancer hospital for appropriate care. During her stay, she underwent staging through a positron emission tomography scan (PET CT), and a bone marrow biopsy was performed before initiating induction chemotherapy. Due to thrombocytopenia (platelet count less than 10,000/μL), intravenous immunoglobulins (IVIG) were initiated.

Within 24 hours of starting the IVIG infusion, she developed hypotension, with a mean arterial pressure (MAP) of 55 mmHg (millimeter of mercury), tachypnea (respiratory rate of 35-38/min), and tachycardia (120 beats per minute). She was afebrile and required oxygen supplementation through a 15-liter non-rebreather mask to maintain an oxygen saturation of around 90%. Due to the severity of her condition, she was urgently transferred to the medical intensive care unit (MICU) within 1-2 hours of clinical deterioration.

**Figure 1.**
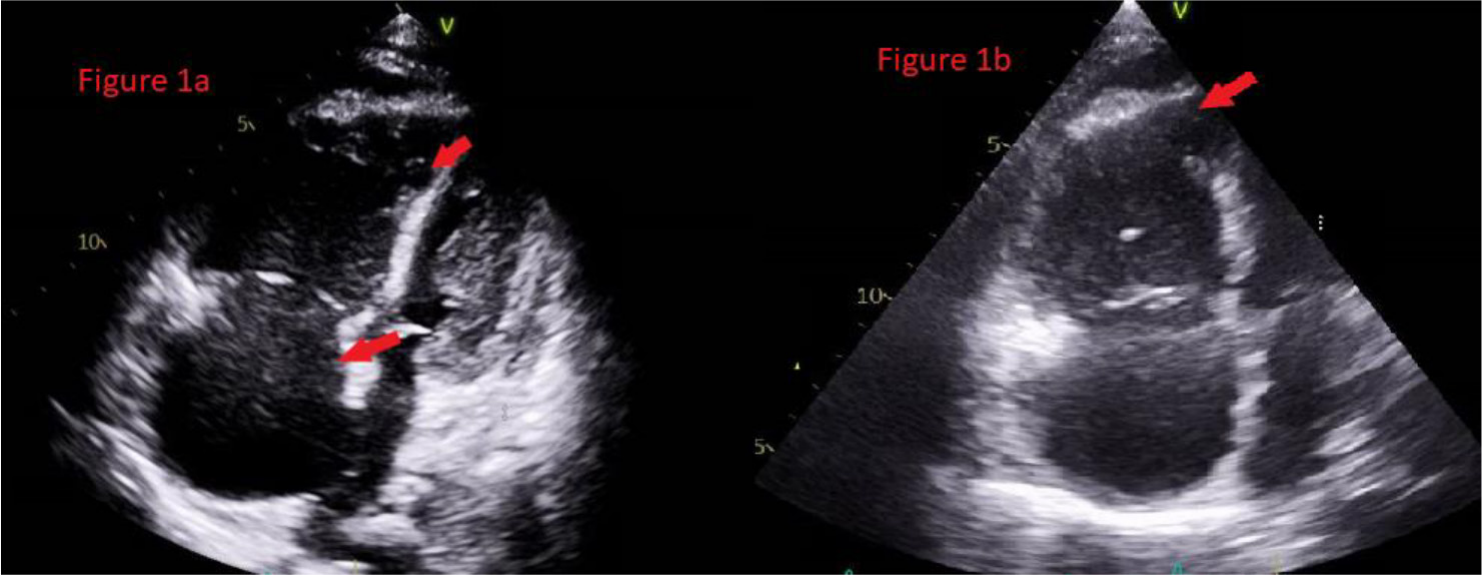
Transthoracic echocardiography of dilated atrium showing early forming Thrombus (a) and dilated right ventricle with McConnell’s sign (b).

Fluid resuscitation was started, and her initial complete blood count revealed hemoglobin of 7.2 g/dL, thrombocytopenia of 6000/μL, and elevated d-dimers. An urgent bedside echocardiogram revealed a severely dilated right ventricle with a strain pattern (as illustrated in Figure 1a), followed by the official echocardiogram that indicated a highly suspected fresh, early-forming thrombus in the inferior vena cava (IVC), approximately 2.0 cm proximal to the inferior vena cava orifice.

Concurrently, the cardiothoracic team was consulted for a potential thrombectomy, but the patient’s deteriorating condition rendered her unfit for surgery. Therefore, an interventional radiologist was consulted for possible interventional radiology-guided thrombectomy or the inferior vena cava filter insertion. Still, she was hemodynamically unstable to be transferred out of the MICU ward. Over the next 3-4 hours, her clinical condition worsened, prompting a drop in her Glasgow Coma Scale (GCS) below 8, requiring endotracheal intubation and mechanical ventilation.

Fifteen minutes after intubation, she developed pulseless ventricular tachycardia. Urgent cardiopulmonary resuscitation (CPR) was started per Advanced Cardiac Life Support (ACLS) protocol, achieving return of spontaneous circulation (ROSC) after one CPR cycle. Blood gases revealed an acidemia with a pH of 6.9, hCO3 of 10 meq/L, CO2 of 80 mmHg, and lactate of 18. Her mean arterial pressure (MAP) barely reached 50 mmHg, necessitating high doses of three different inotropes: noradrenaline, dopamine, and vasopressin.

A repeat bedside transthoracic echocardiography (TTE) showed severe right atrium (RA) and right ventricle (RV) dilation with McConnell’s sign, as depicted in Figure 1b. Subsequently, she experienced two more cardiac arrests with an initial rhythm of pulseless electrical activity (PEA), requiring one cycle of CPR each time to achieve ROSC. The most likely cause of her recurrent cardiac arrest was PE. However, due to severe thrombocytopenia (6000/μL) with a high risk of life-threatening intracranial bleeding, she was deemed unfit for IV thrombolysis.

**Figure 2.**
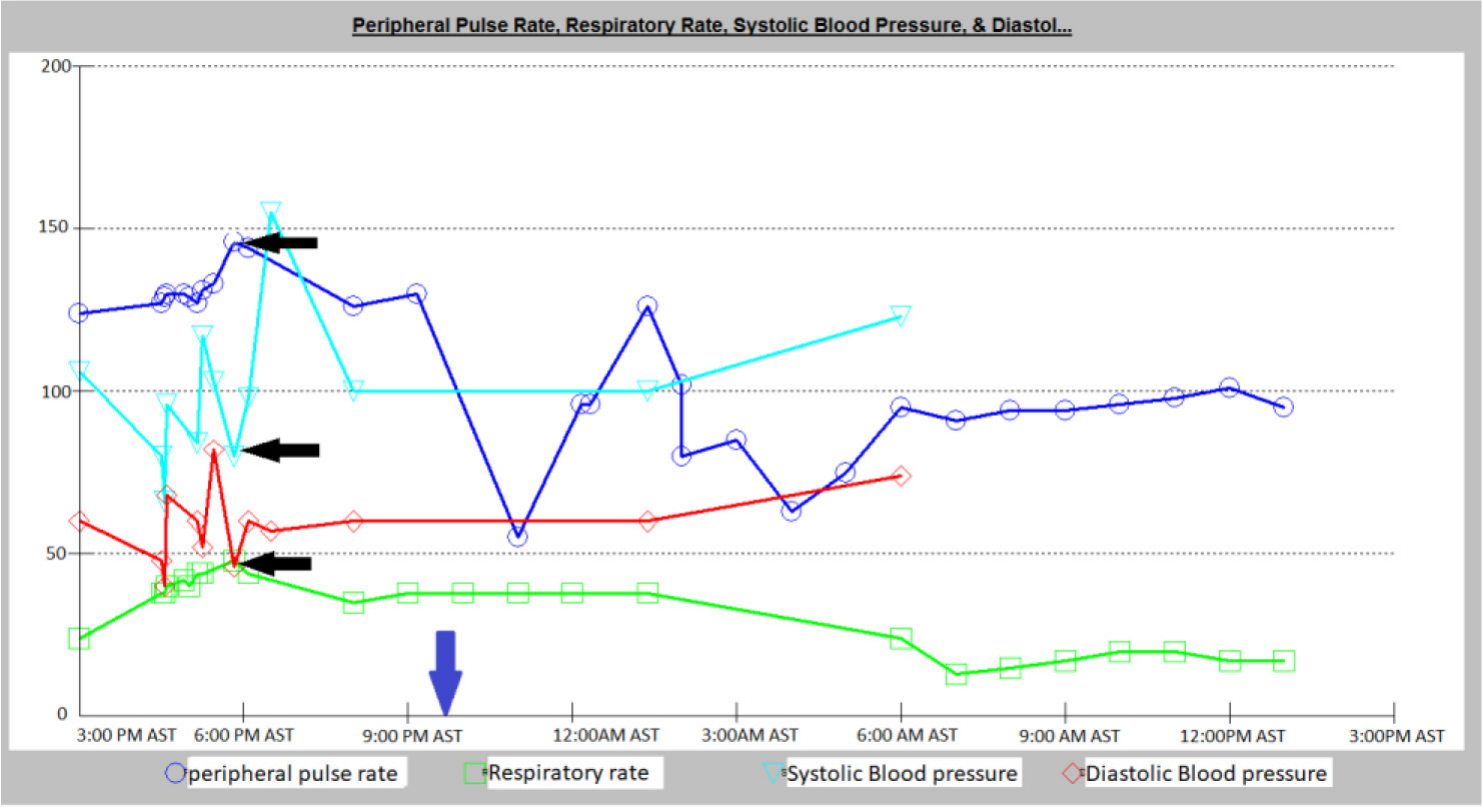
Trend of the physiological parameters with black arrows highlighting unstable vitals during cardiac arrest and subsequent improvement post-Tpa in MICU (~time of Tpa: 9:30 am). (Units: Peripheral pulse rate and respiratory rate units in beats per minute, Blood pressure unit in mmHg).

Transferring her out of the MICU to the interventional radiology department was challenging, and bedside thrombectomy was not an available option. Whether administering or withholding IV thrombolytics in both instances, her prognosis remained uncertain. Consequently, systemic thrombolysis therapy with 50mg of IV Alteplase over 10 minutes was administered as a last measure to save her life. Her blood pressure dramatically improved after 30 minutes of thrombolysis on the same day. Inotropic support was gradually reduced and discontinued within the next 2 hours. Her clinical and laboratory parameters also improved, as detailed in Table 1 and Figure 2.

She experienced mild to moderate fresh bleeding from her nose and mouth and received a transfusion with twelve units of platelets. After the event, her condition improved significantly, leading to successful extubation after two days. She then underwent IVC filter insertion and was transferred to the oncology hospital for lymphoma management. She remained stable during the follow-up period of 4-6 weeks.

## Discussion

High-risk pulmonary embolism (PE) is characterized by a drop in systolic pressure to less than 90 mmHg or a decline in systolic arterial pressure of at least 40 mmHg for at least 15 minutes, which is not caused by new-onset arrhythmias or shock.^2,5^ It carries high mortality; thus, early diagnosis is the key to managing PE. In patients with high-risk PE, 50% die within 30 minutes, 70% die within an hour, and more than 85% die within 6 hours of the onset of symptoms,^5^ emphasizing the need for rapid evaluation and prompt intervention. According to the British Thoracic Society (BTS), major risk factors for PE include major abdominal or pelvic surgery, hip/knee joint replacement, a history of previous venous thromboembolism (VTE), limited immobility as seen in the prolonged bed-bound state, and malignancies.^2^

Definitive diagnosis is made through imaging studies such as bedside TTE, VQ scan, CT angiography, and CTPA.^2^ Management guidelines for high-risk PE include the prompt administration of systemic fibrinolysis/thrombolysis in stable patients without having a risk of bleeding or contraindication or systemic embolectomy/catheter-based thrombectomy in severely hemodynamically compromised patients or those with contraindication to fibrinolysis, which reduces both morbidity and mortality.^6-8^ Certain malignancies are associated with thrombocytopenia and pose a significant risk factor for PE, even in mobile patients.^2,9,10^ The patient, in this case, had lymphoma and severe thrombocytopenia9,10 with a platelet count of less than 10,000/μL.

Furthermore, our patient had high-risk PE, suggested by her underlying lymphoma, elevated d-dimers, and TTE showing RA and RV dilation with McConnell’s sign. She was critically ill, requiring high inotropic support, and experienced multiple consecutive cardiac arrests. In this clinical scenario, mortality was inevitable, and thrombolysis carried an increased risk of major bleeding, such as intracranial hemorrhage. However, after the third arrest, the MICU team decided to give half a bolus dose of the recommended dose of IV Alteplase, i.e., 50mg, as a last resort to saving her life. Her clinical and laboratory parameters improved drastically, leading to successful extubation after two days.

Severe thrombocytopenia of <100,000/mm3 is a contraindication to thrombolysis therapy in stroke patients.^11^ Unfortunately, the literature is deficient, and there is scarce data regarding the management and outcome of high-risk PE associated with thrombocytopenia, commonly associated with autoimmune conditions and immunosuppressive states like malignancies.^12^ A half dose of IV thrombolysis has been reported to show similar beneficial effects and bleeding risk in intermediate high-risk or sub-massive PE; however, implementing this approach in high-risk PE remains questionable.^13,14^

## Conclusion

Malignancy carries a high risk of PE, and certain malignancies, particularly hematological ones, are associated with thrombocytopenia. Thus, in such clinical scenarios, where the management of PE intercepts with thrombocytopenia, decisions become individualized, requiring a delicate balance between benefits and risks. In the presented case, the paper aims to highlight a contemporary approach to managing a life-threatening condition of high-risk PE and thrombocytopenia with a good clinical outcome. Such rare reportable cases bridge the gap in literature deficiency where no standards of care have been established. However, it is imperative to acknowledge the need for further studies to formulate a standardized management approach.

## Statement of Ethics

The patient has consented to publish this case. The study is conducted ethically per the World Medical Association Declaration of Helsinki.

## Approval from the Institutional Research Body

The manuscript completed the review process by the medical research center (MRC) of Hamad Medical Corporation, and the MRC approval number is MRC-04-22-143.

## Conflict of Interest/Disclosure Statement

The authors certify that they have no conflict of interest or affiliations with or involvement in any organization or entity with any financial or non-financial interest in the subject matter or materials discussed in this manuscript.

## Patient Consent

The subject has given verbal and written informed consent to publish the case.

## Data Availability

Authors confirm that all relevant data or information are included in the article and are available via open access platform of this journal.

## Figures and Tables

**Table 1.** Blood gas pH during cardiac arrest, pre-Tpa, and Post-Tpa response.

Blood gas pH	Normal range: 7.350-7.450	Interpretation
During cardiac arrest	6.807	Crit low
Pre-Tpa	7.133	Crit low
Post-Tpa after ~ 2 hours	7.252	low
Post-Tpa after ~ 4 hours	7.400	normal
Post-Tpa after ~ 6 hours	7.441	normal

## References

[bib1] Sadeghi A, Brevetti GR, Kim S, Burack JH, Genovese MH, Distant DA (2005;). Acute massive pulmonary embolism: role of the cardiac surgeon. Tex Heart Inst J.

[bib2] Bĕlohlávek J, Dytrych V, Linhart A (2013;). Pulmonary embolism, part I: Epidemiology, risk factors and risk stratification, pathophysiology, clinical presentation, diagnosis and nonthrombotic pulmonary embolism. Exp Clin Cardiol.

[bib3] QiMin W, LiangWan C, DaoZhong C, HanFan Q, ZhongYao H, XiaoFu D (2020). Clinical outcomes of acute pulmonary embolectomy as the first-line treatment for massive and submassive pulmonary embolism: a single-centre study in China. J Cardiothorac Surg.

[bib4] Iaccarino A, Frati G, Schirone L, Saade W, Iovine E, D’Abramo M (2018;). Surgical embolectomy for acute massive pulmonary embolism: state of the art. J Thorac Dis.

[bib5] Sekhri V, Mehta N, Rawat N, Lehrman SG, Aronow WS (2012). Management of massive and nonmassive pulmonary embolism. Arch Med Sci.

[bib6] Keeling WB, Sundt T, Leacche M, Okita Y, Binongo J, Lasajanak Y (2016). Outcomes After Surgical Pulmonary Embolectomy for Acute Pulmonary Embolus: A Multi-Institutional Study. Ann Thorac Surg.

[bib7] Leacche M, Unic D, Goldhaber SZ, Rawn JD, Aranki SF, Couper GS (2005). Modern surgical treatment of massive pulmonary embolism: results in 47 consecutive patients after rapid diagnosis and aggressive surgical approach. J Thorac Cardiovasc Surg.

[bib8] Kucher N, Goldhaber SZ (2005;). Management of Massive Pulmonary Embolism. Circulation.

[bib9] Tanous O, Dujovny T, Hertzel G, Koren A, Levin C (2020;). Immune Thrombocytopenia Secondary to Hodgkin’s Lymphoma in Children. Isr Med Assoc J.

[bib10] Hauswirth AW, Skrabs C, Schützinger C, Raderer M, Chott A, Valent P (2008). Autoimmune thrombocytopenia in non-Hodgkin’s lymphomas. Haematologica.

[bib11] Fugate JE, Rabinstein AA (2015;). Absolute and Relative Contraindications to IV rt-PA for Acute Ischemic Stroke. Neurohospitalist.

[bib12] Muñoz Tovar RA, Alvarez Perdomo LC, Rojas Molina SM, Salazar SJ (2019). Submassive Pulmonary Thromboembolism in a Patient with Thrombocytopenia: Therapeutic Challenge. Case Rep Crit Care.

[bib13] Kiser TH, Burnham EL, Clark B, Ho PM, Allen RR, Moss M (2018;). Half-Dose Versus Full-Dose Alteplase for Treatment of Pulmonary Embolism. Crit Care Med.

[bib14] Yilmaz ES, Uzun O (2021). Low-dose thrombolysis for submassive pulmonary embolism. J Investig Med.

